# Phase-dependent efficacy of intravenous amniotic mesenchymal stem cells in a rat spinal cord injury model

**DOI:** 10.1186/s13287-026-05018-0

**Published:** 2026-04-24

**Authors:** Yi Qi, Masahito Kawabori, Sho Yamaguchi, Yo Nakahara, Zheng Li, Sumio Ohtsuki, Miki Fujimura

**Affiliations:** 1 Department of Neurosurgery, Faculty of Medicine, Hokkaido University, Kita 15, Nishi 7, Kita-ku, Sapporo, 060-8638 Hokkaido Japan; 2 Regenerative Medicine and Cell Therapy Laboratories, Kaneka Corporation, Kobe, Hyogo Japan; 3 Department of Pharmaceutical Microbiology, Faculty of Life Sciences, Kumamoto University, Kumamoto, Japan

**Keywords:** Spinal cord injury, Amniotic mesenchymal stem cells, Intravenous administration, Therapeutic window, Neuroinflammation, Neutrophils, Macrophages

## Abstract

**Background:**

Spinal cord injury results in profound neurological disability driven initially by primary mechanical damage and subsequently by secondary injury processes characterized by progressive neuroinflammation. Intravenous administration of human amniotic mesenchymal stem cells (MSC) has emerged as a promising therapeutic approach; however, the optimal timing of administration and its relationship to dynamic immune responses remain unclear.

**Methods:**

A rat contusion model of spinal cord injury was used to evaluate the effects of intravenous MSC administration at three post-injury time points: days 1, 3, and 7. Functional and histological assessments were performed for each group. Systemic inflammatory responses were evaluated through blood analysis of neutrophil and macrophage counts, systemic inflammation index (SII), and plasma proteomics. Local immune responses were assessed by quantifying infiltrating immune cells within the injured spinal cord.

**Results:**

The most substantial improvement in locomotor function was observed in the day-1 treatment group, followed by the day-7 group, whereas the day-3 group showed minimal benefit. The day-3 group also demonstrated a trend toward greater lesion length and increased macrophage infiltration 28 days after injury. MSC administration reduced SII in the day-1 and day-7 groups but not in the day-3 group, which instead showed an increased systemic inflammatory response. Analysis of spinal cord tissue demonstrated that MSC treatment on day-1 effectively reduced neutrophil infiltration, which peaks at this time point, while day-7 administration reduced macrophage infiltration during its peak phase. In contrast, MSC administration on day-3 failed to attenuate either neutrophil or macrophage accumulation. Plasma proteomic profiling revealed enhanced complement and coagulation pathway activation specifically on day-3.

**Conclusions:**

The therapeutic efficacy of intravenously administered MSC is highly dependent on the timing of intervention. Optimal benefit is achieved when treatment coincides with peak activation of a dominant target immune cell population and avoids the peak of complement and coagulation signaling. These findings support a phase-matched therapeutic strategy to maximize MSC effectiveness following spinal cord injury.

**Supplementary Information:**

The online version contains supplementary material available at 10.1186/s13287-026-05018-0.

## Introduction

Spinal cord injury (SCI) is a highly disabling disorder of the nervous system. Patients frequently experience enduring deficits in motor, sensory, and autonomic function and bear a substantial socioeconomic burden [1, 2]. Beyond the initial mechanical insult, a subsequent “secondary injury” cascade—including ischemia, excitotoxicity, oxidative stress, and a dynamically evolving neuroinflammatory response—drives progressive tissue loss and functional deterioration. After SCI, the inflammatory milieu evolves rapidly: microglial activation leads to neutrophils infiltration, followed by infiltration of monocytes/macrophages, while astroglial and fibrotic scarring develops over days to weeks. Accordingly, strategies that modulate these secondary processes, particularly those that remodel the immune microenvironment, constitute a central focus of contemporary research in neuroprotection and neural repair [3, 4].

Mesenchymal stem/stromal cells (MSC) have emerged as promising candidates for SCI therapy owing to their low immunogenicity, secretion of trophic and anti-inflammatory factors, release of extracellular vesicles, and capacity to influence glial and immune cell phenotypes to promote neuroprotection and plasticity [5–7]. Among MSC sources, amniotic MSCs (AMSCs) have attracted special interest because of their perinatal origin, robust paracrine activity, and favorable safety profile [8, 9]. Although a substantial fraction of intravenously administered cells undergoes pulmonary first-pass entrapment,^10^ systemic immunomodulation, altered leukocyte homing/migration, and indirect actions on the injured cord can nonetheless yield functional benefits across multiple animal models [11–13].

Prior studies adopting MSC for SCI have produced inconsistent findings, with some supporting very early treatment and others suggesting that delayed intervention may be equally effective [14, 15]. However, direct head-to-head comparisons of different dosing time points using the same cell product, route, and dose are scarce, and the mechanistic relationship between administration timing and the evolving inflammatory-cell landscape has not been systematically elucidated. To address this gap, this research compared IV AMSC administration on day 1, day 3, and day 7 after injury in an adult Sprague–Dawley rat contusion SCI model. We assessed locomotor recovery, quantified lesion histopathology, and evaluated inflammatory responses at both peripheral and local tissue levels. To elucidate possible mechanisms, we performed histological analyses of immune cells within the spinal cord at the corresponding time points to test whether therapeutic efficacy aligns with the presence of a “targetable dominant inflammatory cell” at each stage.

## Methods

Detailed procedures are described in the supplementary methods.

### Experimental ethics and design

Animal protocols were approved by the Animal Studies Ethics Committee of the Hokkaido University Graduate School of Medicine. Approval number: 21–0123. All experimental procedures were conducted in accordance with the Institutional Guidelines for Animal Experimentation, the Guidelines for Proper Conduct of Animal Experiments issued by the Science Council of Japan, and the ARRIVE guidelines 2.0.

In total, 126 rats were used, including animals reserved for potential attrition and those excluded according to pre-specified criteria. Nine-week-old adult female Sprague–Dawley (SD) rats (CLEA Japan, Inc., Japan) were allocated to four groups: Vehicle, AMSC Day-1 administration (D1 group), AMSC Day-3 administration (D3 group), and AMSC Day-7 administration (D7 group). To rigorously control injection-related confounders, all animals received tail-vein injections on post-injury days 1, 3, and 7 with volume-matched solutions as follows: Vehicle received sterile saline on days 1, 3, and 7; D1 received AMSC on day 1 and saline on days 3 and 7; D3 received AMSC on day 3 and saline on days 1 and 7; and D7 received AMSC on day 7 and saline on days 1 and 3. The exact number of experimental units per group and per analysis is detailed in the corresponding figure legends and methods sections. The sample size was determined based on preliminary experiments. This number was considered sufficient to detect biologically meaningful differences while minimizing animal use in accordance with ethical guidelines.

### Rat spinal cord injury model

Rats were anesthetized with 2–5% isoflurane in 30% O₂/70% nitrous oxide (N₂O), and core temperature was maintained at 36.5–37.5 °C with a heating pad. Thoracic (T6–T7) clip-compression SCI was produced by extradural pinching with a modified aneurysm clip (MIZUHO, Tokyo, Japan) for 1 min, as described previously [9, 16–18]. At 24 h post-injury, model success was assessed using the Basso–Beattie–Bresnahan (BBB) score; animals with BBB ≠ 0 were excluded from subsequent studies. Humane endpoints were predefined prior to study initiation. Animals were monitored daily for signs of pain or distress, including but not limited to body weight loss exceeding 20%, reduced mobility, abnormal posture, and self-mutilation. Animals meeting these criteria were humanely euthanized under deep anesthesia induced with 5% isoflurane in a gas mixture of 30% O₂ and 70% N₂O, followed by cervical dislocation, in accordance with institutional guidelines and approved ethical protocols. Cage positions within the animal facility were regularly rotated to reduce the influence of environmental variables such as light, temperature, and noise. In addition, the investigator performing the SCI was independent.

### AMSC preparation and cell administration

Frozen human AMSC vials were supplied by Kaneka Corporation (Osaka, Japan) and prepared as previously reported [9, 17, 19, 20]. After 6-day recovery cultivation, the AMSC was diluted with saline to a target concentration of 1 × 10⁶ cells/mL. Animals were randomly assigned to experimental groups and received either 1 mL of AMSC suspension or vehicle (normal saline) via the lateral tail vein at an injection rate of 1 mL/min. Group allocation was implemented as follows at each stage of the experiment. During group allocation and treatment administration, animals were randomly assigned to experimental groups. The investigator responsible for intravenous administration was aware of the group allocation at the time of injection, as required to deliver the correct treatment; this investigator was not involved in subsequent behavioral scoring, histological quantification, or data analysis. Animals were then euthanized at designated time points for subsequent histological evaluation [21, 22].

### Neurological and histological evaluation

The primary outcome measure was open-field locomotion function, assessed using the 21-point Basso–Beattie– Bresnahan (BBB) scale once weekly until 28 days post-SCI, as previously described [9, 17, 23]. Klüver–Barrera (KB) myelin/Nissl double staining was performed 28 days after SCI to evaluate lesion segment length based on the continuous absence of Luxol fast blue (LFB) staining as previously reported [9, 17, 24]. CD68 (Bio-Rad, Cat. 159320, 1:1000), Iba 1 (Fujifilm Wako, Cat. CAP4688, 1:1500), MPO (Abcam, ab208670, 1:1000) staining were performed to characterize immune-cell regulation in the spinal cord after SCI and at defined treatment time points [25–27]. For outcome assessment, all behavioral evaluations (BBB scoring), histological staining, image acquisition, and quantitative analyses were performed by investigators who were blinded to group allocation.

### Complete blood count (CBC) and systemic immune-inflammation index (SII)

Rats were randomized into five groups (*n* = 5/group; Control group, vehicle group, D1 group, D3 group and D7 group). Longitudinal blood sampling was scheduled at D1, D2, D3, D4, D7, D8, and D14 (a.m.); AMSC (or vehicle) injections were performed D1 (p.m.), D3 (p.m.), and D7 (p.m.) as described above. 100 µL of whole blood was collected by a tail-vein nick (right lateral tail vein), and were analyzed within 2 h of collection. CBC and multi-part leukocyte differentials were measured on an automated hematology analyzer (Sysmex XN-2000, WDF channel; Sysmex Corporation, Kobe, Japan) configured for rat blood. The systemic immune-inflammation index (SII) was computed from absolute counts as SII = (platelet count × neutrophil count)/lymphocyte count [28].

### Blood plasma proteomics

Data Independent Acquisition mass spectroscopy (DIA-MS) was performed for blood plasma at days 1, 3, and 7 after SCI to profile post-injury plasma proteome dynamics. ZenoTOF 7600 mass spectrometer (Sciex, Framingham, MA, USA) coupled with the Dionex Ultimate 3000 RSLCnano System (Dionex, Sunnyvale, CA, USA) was used for DIA-MS as previously described [29]. The peptides and proteins were identified and quantified using DIA-NN 1.8.1 with UniProt rat reference proteome data and filtering at a false discovery rate of < 1%. Normalized protein-abundance matrices were analyzed in RNAseqChef https://imeg-ku.shinyapps.io/RNAseqChef_imeg/ [30]. Differential expression analysis between the 1, 3, and 7 days group after injury was conducted using the limma package for normalized count data. Given the exploratory nature of this study, proteins with false discovery rate (FDR) less than 0.1 were considered as significantly differentially expressed; among these, fold change ≥ 1.20 defined upregulation and ≤ 0.83 defined downregulation. Differentially expressed proteins were submitted to STRING v12.0 (https://string-db.org/) for protein–protein interaction network construction and enrichment [31].

### Statistics

All numerical data are expressed as mean ± standard error of the mean (SEM) or as median with interquartile range (IQR), as appropriate. Statistical significance of the differences between the means was determined using the unpaired two-tailed Student’s t-test and Welch’s t-test for two groups and one-way analysis of variance (ANOVA) followed by Tukey’s test or comparisons with a control using Dunnett’s method for more than two groups. Proteomics-specific multiple-testing is described in the proteomics Methods. All statistics analyses were performed using GraphPad Prism 9 (GraphPad Software, San Diego, CA, USA). *p* < 0.05 is considered statistically significant.

## Results

**Fig. 1:**
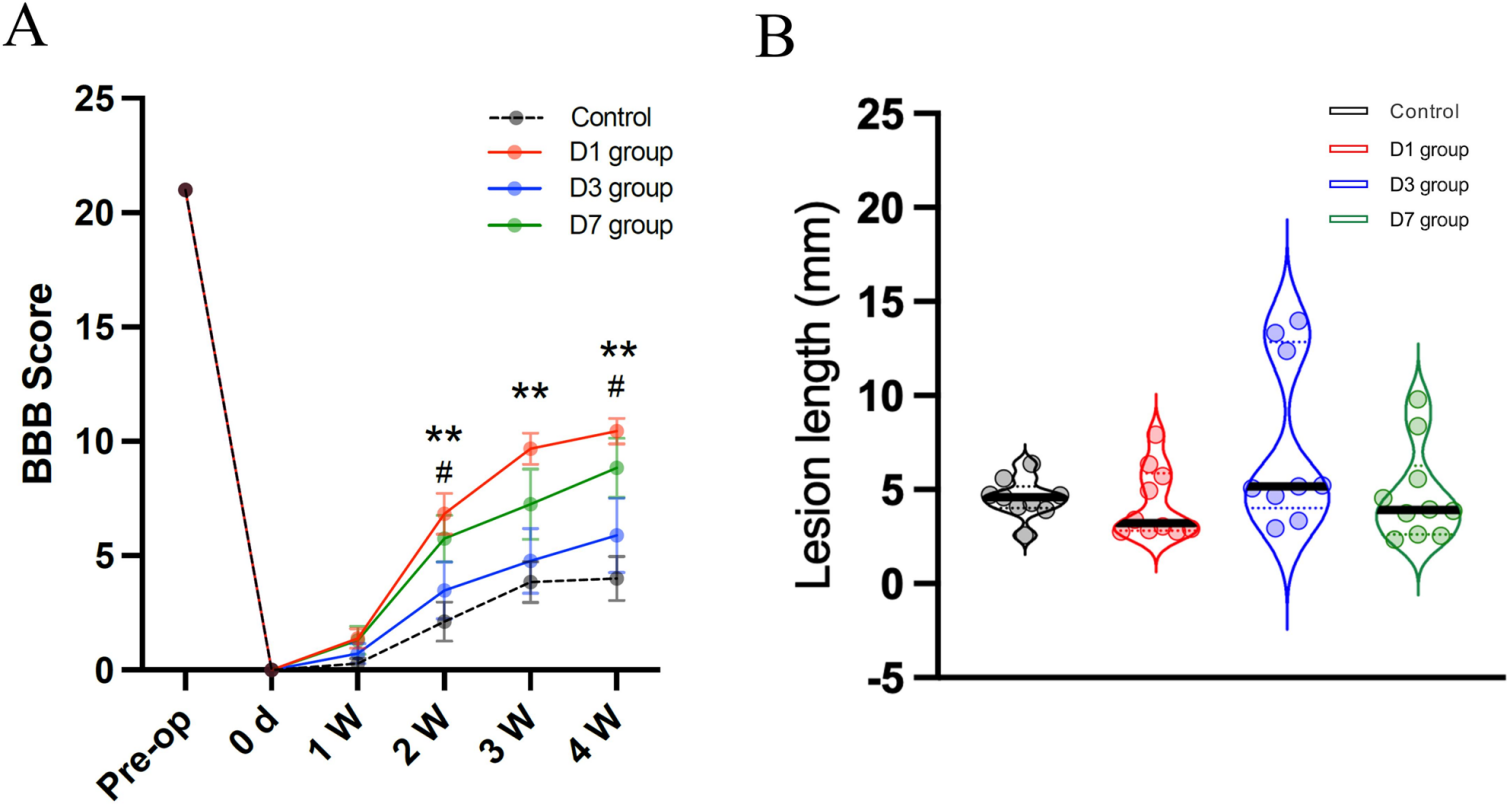
Functional assessment and damaged lesion size after SCI. **A** BBB scores demonstrated that the day-1 (D1) group exhibited significantly greater functional improvement than the control group at weeks 2, 3, and 4. The day-7 (D7) group also showed significant improvement at weeks 2 and 4. In contrast, the day-3 (D3) group did not differ significantly from controls at any evaluated time point. **B** Lesion length analysis revealed comparable tissue damage in the D1 (4.3 ± 0.6 mm) and D7 (4.7 ± 0.8 mm) groups relative to controls (4.5 ± 0.6 mm). Although not statistically significant, the D3 group (7.3 ± 1.4 mm) exhibited a greater lesion length than the control group. Control (*n* = 9), D1 (*n* = 10), D3 (*n* = 9), D7 (*n* = 10). *^, #^*P* < 0.05, **P < 0.01

A total of 126 rats were used in this study, including animals allocated for potential attrition and those excluded according to pre-specified criteria. Open-field BBB scoring revealed that D1 group (AMSC administration at day 1 and saline administration at day 3 and 7) exhibited significantly greater functional improvement than controls at week 2 (*p* < 0.01), week 3 (*p* < 0.01), and week 4 (*p* < 0.01). The D7 group (AMSC administration at day 7 and saline administration at day 1 and 3) also demonstrated significant improvement at week 2 (*p* = 0.049) and week 4 (*p* = 0.02). In contrast, the D3 group (AMSC administration at day 3 and saline administration at day 1 and 7) did not differ from controls at any evaluated time point (e.g., week 4, *p* = 0.55) (Fig. 1A).

**Fig. 2:**
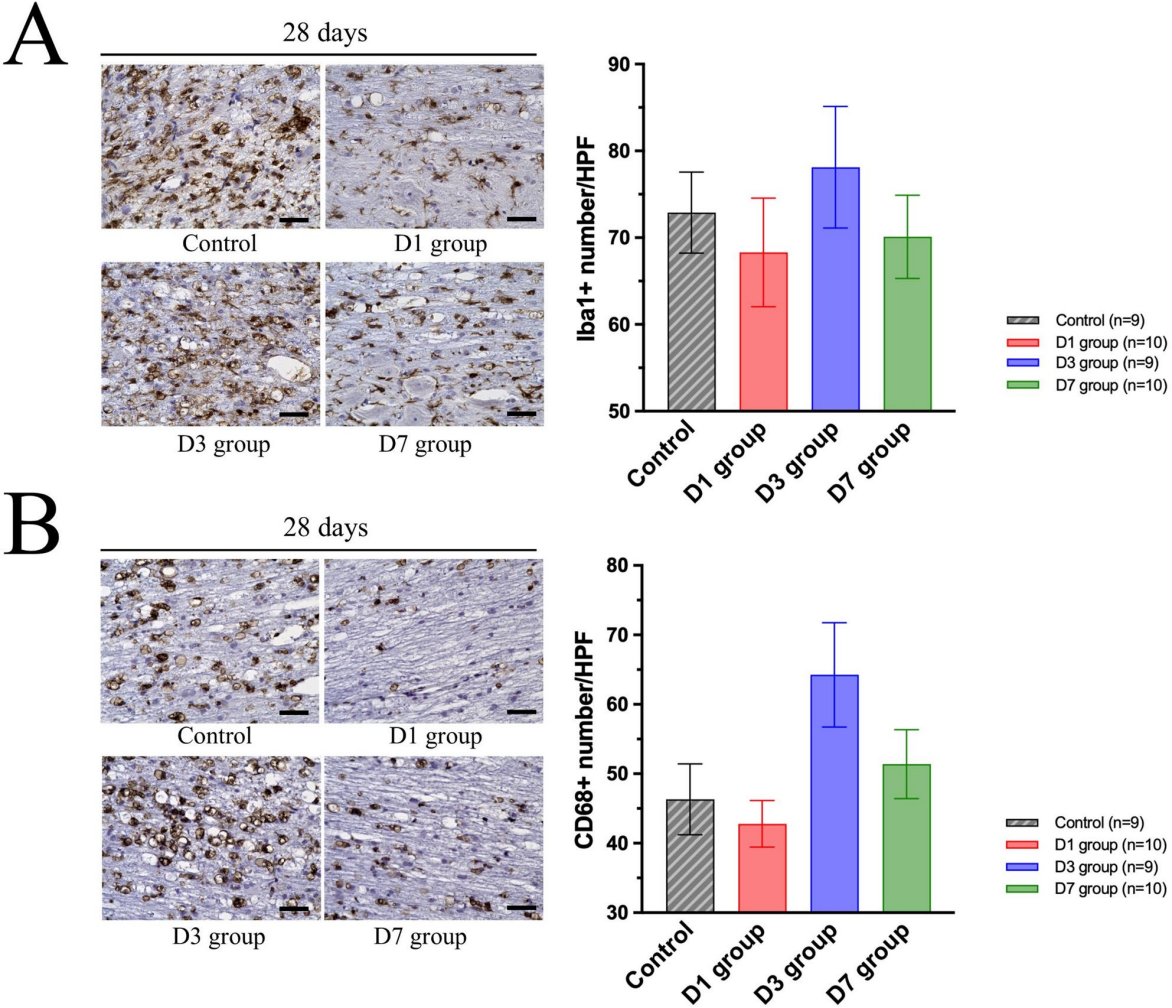
Immune response after cell administration. **A** Representative Iba1 immunohistochemistry at day 28. D1 group exhibited the fewest activated cell in the damaged area (decreased by 6.3% compared with control), followed by that of D7group (decreased by 3.8%). On the other hand, D3 group (increased of 7.2%) showed higher cell number compared to control group. **B** Representative CD68 immunohistochemistry at day 28. D1 group (decreased by 7.6%) showed the fewest cells followed by control cells, while D7 showed slightly higher cells (increased of 11.0%), and D3 group higher cell infiltration (increased of 38.6%), the one-way ANOVA indicated a significant overall group effect (*p* = 0.04) (Fig. 2B). Scale bar = 50 μm. Data are mean ± SEM

Spinal cord pathology was further quantified by measuring damaged lesion length. Notably, the D1 (4.3 ± 0.6 mm) and D7 (4.7 ± 0.8 mm) groups demonstrated lesion lengths comparable to the control group (4.5 ± 0.3 mm). Although not statistically significant, the D3 group exhibited substantially greater tissue damage (7.4 ± 1.4 mm) (Fig. 1B). Similar trends were also found regarding the immune responses at spinal cord 4 weeks after injury. Iba-1 staining, representing activated microglia, showed that D1 group exhibited the fewest activated cell in the damaged area (decreased by 6.3% compared with control), followed by that of D7 group (decreased by 3.8%). On the other hand, D3 group showed higher cell number compared to control group (increased of 7.2%) (Fig. 2A). CD68 staining, representing infiltrated macrophage, showed comparative result that D1 group showed the fewest cells followed by control cells (decreased by 7.6%), while D7 showed slightly higher cells (increased of 11.0%), and D3 group higher cell infiltration (increased of 38.6%), the one-way ANOVA indicated a significant overall group effect (*p* = 0.04) (Fig. 2B).

**Fig. 3:**
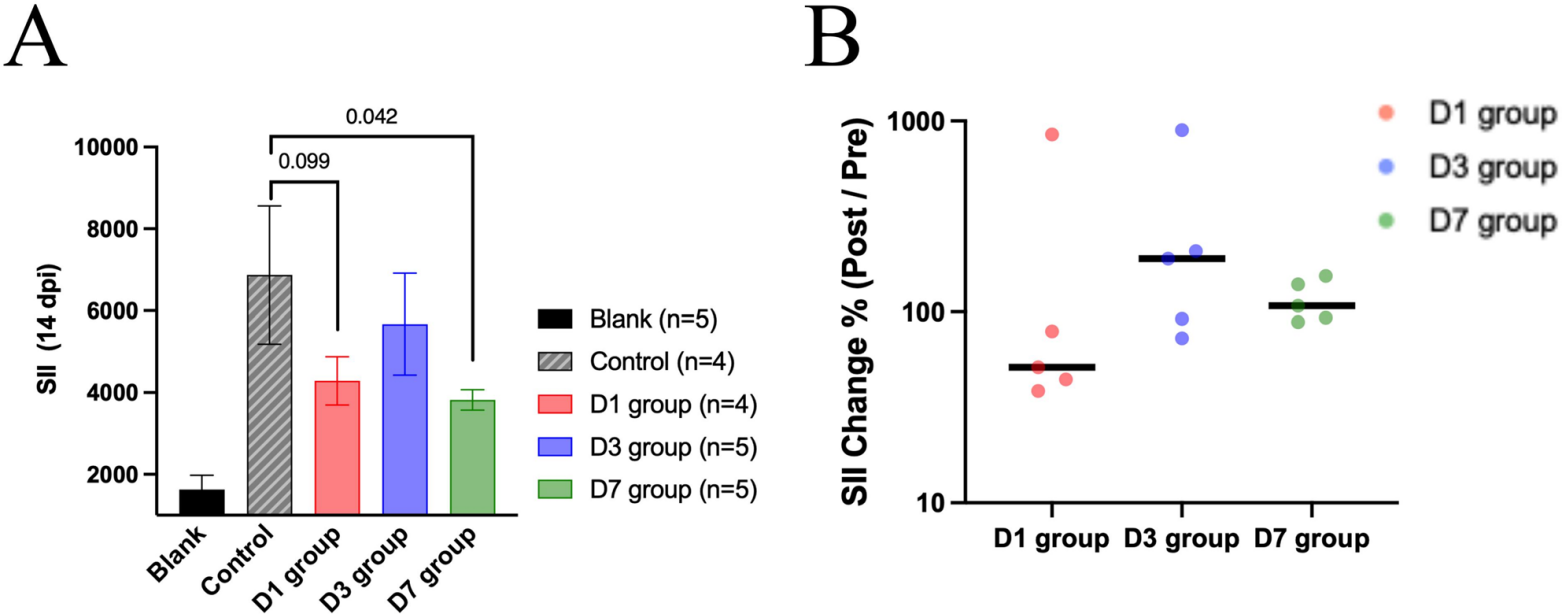
Systemic inflammatory index (SII) after SCI and cell administration. **A** SII at day 28 was reduced in the day-1 (D1) and day-7 (D7) groups, whereas SII levels in the day-3 (D3) group were comparable to those in the control group (*p* = 0.099, *p* = 0.04, and *p* = 0.29, respectively). **B** Changes in SII before and after cell administration were analyzed at each treatment time point. The SII ratio in the D1 group (day 2/day 1) showed a marked reduction (51.4%, interquartile range [IQR] 41.6–464.3). In contrast, the SII ratio in the D7 group (day 8/day 7) remained relatively stable (107.5%, IQR 90.8–146.7), whereas a pronounced increase in systemic inflammation was observed in the D3 group (day 4/day 3; 190.1%, IQR 82.4–551.5)

The systemic inflammatory response following cell administration was further evaluated. At day 28, the SII was reduced in the D1 and D7 groups, whereas SII levels in the D3 group were comparable to those in the control group (*p* = 0.099, *p* = 0.04, and *p* = 0.29, respectively) (Fig. 3A). Changes in SII before and after cell administration at each time point were subsequently analyzed. The SII ratio for the D1 group (day 2/day 1) showed a marked reduction (51.4%, IQR 41.6–464.3). In contrast, the SII ratio for the D7 group (day 8/day 7) remained relatively stable (107.5%, IQR 90.8–146.7), whereas a pronounced exacerbation of systemic inflammation was observed in the D3 group (day 4/day 3; 190.1%, IQR 82.4–551.5) (Fig. 3B). Collectively, these findings indicate that the systemic immune response to stem cell administration is highly dependent on treatment timing. Administration during the acute (day 1) and subacute (day 7) phases attenuated systemic inflammation, whereas treatment on day 3 was associated with exacerbation of the inflammatory response.

**Fig. 4:**
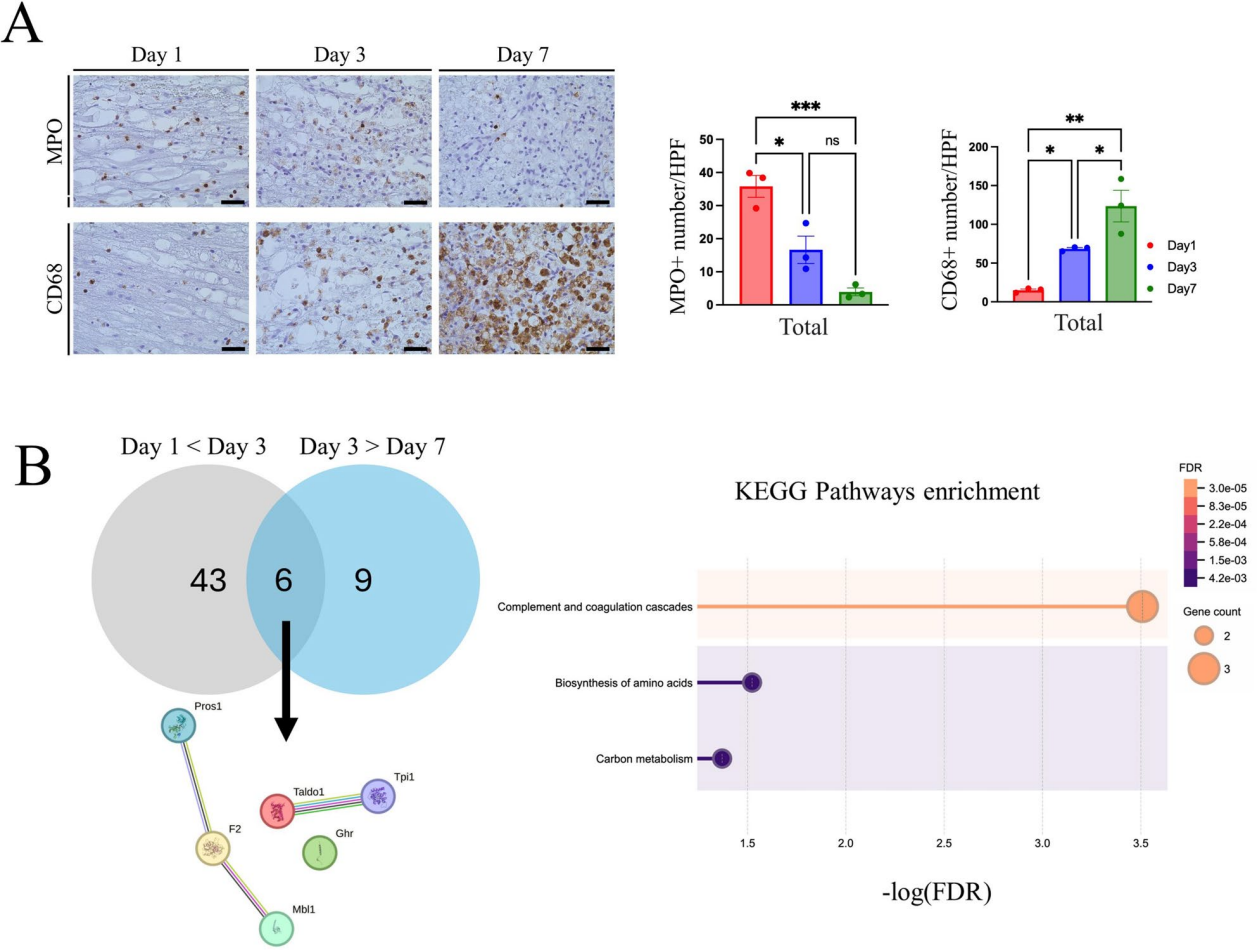
Temporal dynamics of dominant immune cell populations after SCI. **A** Representative immunohistochemistry at post-injury days 1, 3, and 7: MPO (neutrophils) and CD68 (macrophages). Quantification of MPO⁺ cells per high-power field (HPF): neutrophil burden peaks at D1 and declines thereafter. Quantification of CD68⁺ cells per HPF: macrophage burden peaks at D7. **B** Plasma proteomics: pathway activity/enrichment scores show higher complement and coagulation cascade activity at D3 versus D1 and D7. Scale bar = 50 μm. Data are mean ± SEM. *P < 0.05, ** *P* < 0.01, *** *P* < 0.001

**Fig. 5:**
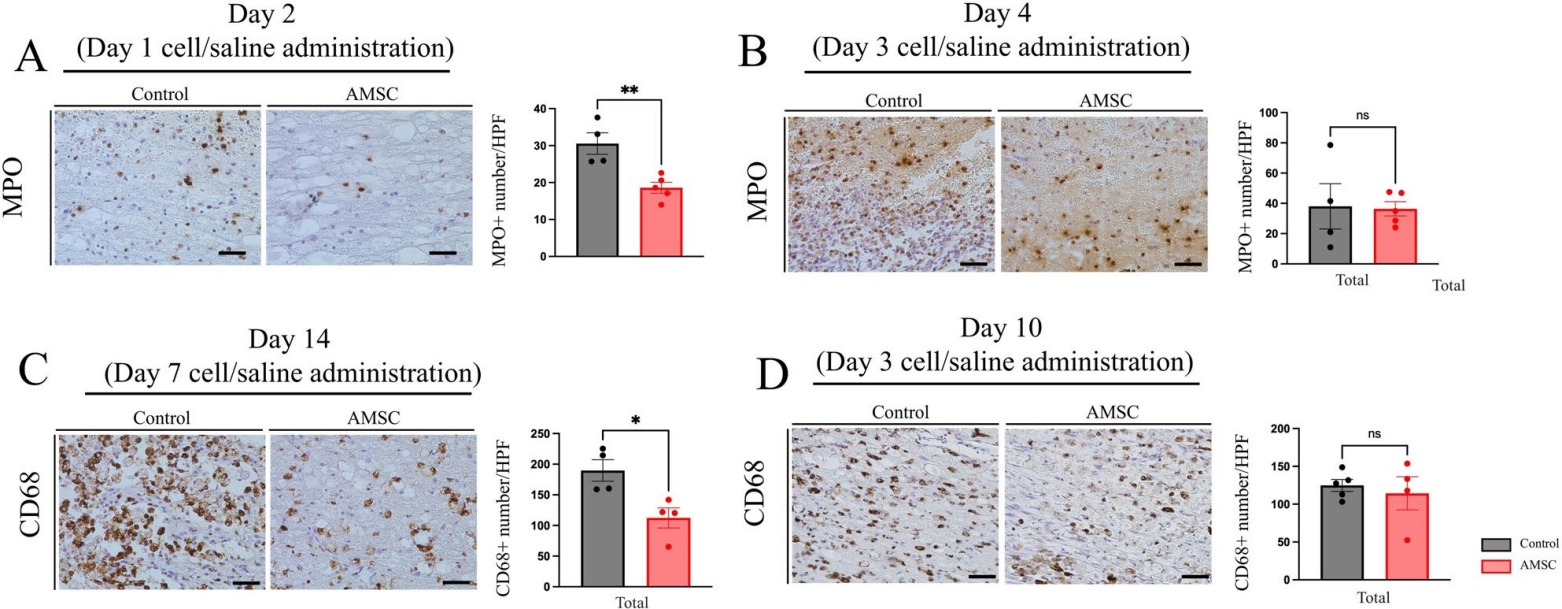
Phase-specific immunomodulatory effects of AMSC administration following spinal cord injury. **A** AMSC administration on day 1 significantly reduced myeloperoxidase (MPO)–positive neutrophil infiltration into the spinal cord at day 2. **B** No significant difference in MPO-positive neutrophil infiltration was observed between AMSC and saline-treated groups when administration occurred on day 3, as assessed at day 4. **C** AMSC administration on day 7 significantly decreased CD68-positive macrophage infiltration into the spinal cord at day 8. (D) No significant difference in CD68-positive cell infiltration was detected between AMSC and saline-treated groups when administration occurred on day 3, as assessed at day 4. Scale bar = 50 μm. *P < 0.05, **P < 0.01

To elucidate the mechanisms underlying the differential efficacy observed across administration time windows, we first evaluated infiltration of systemic immune cells into the spinal cord—representing the local inflammatory response—at post-injury days 1, 3, and 7 in the absence of cell administration. Myeloperoxidase (MPO)–positive activated neutrophils peaked on day 1 and declined thereafter (Fig. 4A), whereas CD68-positive activated macrophages progressively increased and reached a maximum on day 7, indicating macrophage predominance at this later stage. On day 3, neither cell population predominated; instead, both markers were present at intermediate levels, suggesting a transitional inflammatory state. Consistent with these tissue-level findings, plasma proteomic analyses demonstrated that proteins upregulated on day 3, relative to both days 1 and 7, were enriched for complement and coagulation cascade pathways, indicating heightened activation of these systems at this time point (Fig. 4B). Collectively, these data indicate a temporal shift in dominant immune targets—from neutrophils on day 1 to macrophages on day 7—with no clearly dominant population on day 3, providing a plausible mechanistic explanation for the observed efficacy ranking of intravenous AMSC treatment (day 1 > day 7 > day 3). To validate this phase-specific immunomodulatory mechanism, we next assessed neutrophil (MPO) and macrophage (CD68) infiltration following cell administration. Neutrophil infiltration was significantly reduced at day 2 when AMSCs were administered on day 1 (Fig. 5A), whereas no significant change in MPO-positive cells was observed when administration occurred on day 3 (Fig. 5B). Similarly, CD68-positive macrophage infiltration was significantly decreased when cells were administered on day 7 (Fig. 5C), whereas no significant effect was observed following administration on day 3 (Fig. 5D). These findings indicate that AMSC treatment effectively attenuates the neutrophil-dominant inflammatory response when administered on day 1 and the macrophage-dominant response when administered on day 7. In contrast, consistent with a transitional inflammatory milieu lacking a dominant, targetable immune population, AMSC administration on day 3 failed to modulate either neutrophil or macrophage infiltration.

## Discussion

In this study, we conducted a head-to-head comparison of the therapeutic efficacy of intravenously infused human AMSCs administered at different post-injury time points after SCI, using the same animal model, route, and dose across arms. Three principal findings emerged. (i) Functional outcomes exhibited a clear time-dependence (D1 > D7 > D3; D1/D3/D7 denote days post-injury). (ii) The efficacy ranking paralleled the dominant immune-cell composition within the lesion: MPO⁺ neutrophils predominated at D1, CD68⁺ macrophages/microglia predominated at D7, and D3 represented a transitional phase with both populations at intermediate levels. (iii) Mechanistic validation showed that D1 dosing reduced neutrophil burden, whereas D7 dosing reduced macrophage burden; in parallel, the peripheral systemic immune-inflammation index at D14 was decreased in both the D1 and D7 groups relative to controls, indicating a central–peripheral coupling of AMSC-mediated immunomodulation. Collectively, these data support a “phase-matching” concept: AMSC efficacy is maximized when the dosing window overlaps the period in which the predominant, modifiable immune target cell population is present.

Prior work on the benefits of mesenchymal stromal cells (MSCs) in spinal cord injury (SCI) has largely framed the question in terms of “whether they work” or “the earlier, the better,” yet heterogeneity in models, cell sources, and dosing has yielded inconsistent conclusions [5, 9, 15, 32, 33]. The distinctive contribution of the present study is to align “efficacy differences” with the “temporal phases of immune-cell dominance” within a single framework: D1 dosing coincides with the neutrophil peak and achieves the greatest functional benefit; D7 dosing aligns with the macrophage activation phase and yields an intermediate benefit; whereas D3 performs poorly, likely because the immune landscape has not yet consolidated into a single dominant population. These findings provide direct evidence that a single intravenous MSC treatment is not simply a matter of earlier being better, but rather of synchronizing administration with the prevailing, plastic immune cell target. They may also help reconcile discordant reports in the literature—namely, that very early intervention is effective, delayed dosing can still confer benefit, yet certain intermediate windows perform suboptimally [11, 14, 34–37]. Although lesion length in the D3 group appeared numerically greater than in controls, variability was increased during this transitional inflammatory phase, resulting in a wide confidence interval. Thus, while overt worsening was not statistically confirmed, the absence of function improvement and lack of immune modulation support the interpretation of D3 as a non-permissive window rather than a definitively harmful one.

AMSCs exhibit low immunogenicity and robust paracrine activity, and can alter immune-cell fate and phenotype via mediators such as PGE2, TSG-6, and IL-10, as well as through extracellular vesicles (EVs) [17, 38–40]. At D1, neutrophils predominate and drive oxidative stress and proteolytic injury; systemic immunomodulation by AMSCs—including first-pass pulmonary sequestration followed by “remote education” of circulating granulocytes/monocytes—can more rapidly suppress neutrophil recruitment, activation, or NET formation (NETosis), [41] thereby mitigating acute secondary damage [10, 42–44]. This is consistent with our observed decline in MPO⁺ cells and the subsequent reduction of the SII. By D7, macrophages/microglia become the principal effectors; AMSCs may remodel cytokine networks and metabolic programs to bias polarization from pro-inflammatory (M1-like) toward reparative (M2-like) states, thereby improving the tissue microenvironment and its plasticity/remodeling potential [45, 46]. In contrast, at D3, complement and coagulation cascades are more active in plasma proteomics, indicating an intermediate state characterized by inflammatory crosstalk and cascade amplification. Post-traumatic hypercoagulability has been widely recognized as a critical complication, typically peaking approximately 72 h after injury and subsiding within five days. Although the precise mechanisms underlying trauma-associated hypercoagulopathy remain incompletely defined, compliment and activated protein C is believed to play a central regulatory role in coagulation pathway activation [47]. In this window, and positive feedback among complement–coagulation–myeloid axes may be present. Liao et al. demonstrated that intravenous MSCs can elicit a tissue factor–mediated coagulation response, characterized by platelet consumption, prolongation of prothrombin time and activated partial thromboplastin time, and disseminated microthrombi; they further showed that this response increases pulmonary embolization and clearance, thereby diminishing biodistribution to target organs. Administration of heparin (400 U/kg) abrogated MSC-induced coagulation reduced first-pass pulmonary sequestration, and enhanced trafficking to and persistence within target tissues, ultimately improving efficacy [48]. In this environment, we speculate that intravenously delivered AMSCs may be exposed to complement-mediated cytotoxicity and enhanced instant blood-mediated inflammatory reactions (IBMIR) [49]. Unlike Day 1 and Day 7, when a dominant immune-cell population is present and selectively modifiable, Day 3 lacks a clearly prevailing targetable compartment and instead reflects inflammatory cross-talk among neutrophils, macrophages, complement, and coagulation pathways. Thus, the limited efficacy observed at this time point may reflect a temporally non-permissive immune landscape rather than intrinsic ineffectiveness of the cells.

Our results support a unified immunological-balance model that explains the timing-dependent therapeutic effects of intravenous AMSC administration after spinal cord injury. In this model, the treatment outcome is determined by the weighted interaction among three biological processes: (1) the infiltration of peripheral immune cells—such as neutrophils in the acute phase and macrophages in the subacute phase—together with the beneficial regulatory effects exerted by AMSC specifically during these infiltration windows; (2) xenogeneic immune rejection elicited by administered human cells; and (3) activation of the complement and coagulation cascades. Accordingly, at Day 1, peripheral neutrophil infiltration is at its peak, and AMSC can effectively modulate excessive inflammation during this window, while xenorejection remains moderate and complement activation minimal, resulting in an overall positive balance. At Day 3, complement and coagulation responses reach their maximum levels, and this dominant negative influence outweighs the immunomodulatory effects of AMSC, shifting the overall balance toward a neutral state. By Day 7, macrophage infiltration increases again and tends toward a reparative phenotype, while complement activity declines; under these conditions, AMSC-mediated macrophage modulation becomes predominant, restoring a positive immunological balance. Although our data demonstrate phase-specific attenuation of neutrophils and macrophages, the present study cannot definitively distinguish between direct lesion-site interaction and systemic immunomodulatory reprogramming. Given the known pulmonary first-pass effect of intravenously delivered MSCs, it is likely that AMSC exert their therapeutic influence predominantly through systemic signaling, including paracrine mediator release and re-education of circulating myeloid populations, which subsequently alters immune-cell trafficking to the injured cord. Our recent work further supports this concept, that Morishima et al. demonstrated that intravenous administration of MSC-derived exosomes attenuated spinal cord injury by suppressing neutrophil extracellular trap (NET) formation via exosomal miR-125a-3p, highlighting a systemic neutrophil-regulatory mechanism [17]. Such findings suggest that MSC-based therapies may modulate innate immune responses not through direct blockade of lesion-resident cells, but by reshaping peripheral neutrophil activation states before their infiltration into injured tissue. While we used a fixed dose of 1 × 10⁶ cells based on prior optimization and translational consistency, it remains possible that higher doses might partially overcome the Day 3 inflammatory barrier. However, given the observed complement and coagulation activation during this phase, dose escalation may increase cell clearance and exacerbate instant blood-mediated inflammatory reactions. Therefore, the Day 3 window may represent an environment-restricted phase rather than a purely dose-limited one.

Because immune rejection would be substantially reduced in clinical allogeneic settings compared with xenogeneic rodent models, this immunological-balance model suggests that the optimal timing for cell administration should fall within phases characterized by prominent peripheral immune-cell infiltration and relatively low complement/coagulation activation, thereby maximizing the immunoregulatory effects of AMSC. This framework provides a theoretical basis for determining the therapeutic window in future early-phase clinical trials for acute SCI. Although temporal scaling may differ between rodents and humans, the progression of inflammatory phases after SCI follows a broadly conserved pattern across species, beginning with early neutrophil predominance and transitioning toward macrophage-dominated responses. In rodents, these phases correspond approximately to post-injury days 1 and 7, respectively. In humans, the hyper-acute neutrophilic phase typically occurs within the first 24–48 h, while macrophage-driven remodeling becomes prominent during the early subacute period (approximately 5 days – 2 weeks after insult). Given the relatively low immunogenicity of allogeneic AMSCs in clinical settings, cellular persistence in humans may exceed that observed in xenogeneic rodent models, potentially contributing to improved therapeutic outcomes in patients. Importantly, our findings suggest that therapeutic efficacy depends more on alignment with the dominant immune phase. Thus, immune-phase–guided administration, potentially informed by circulating biomarkers or imaging-based inflammatory profiling, may represent a rational strategy for clinical MSC trial design.

This study has several limitations. First, we did not perform in vivo tracking of transplanted cells at the different administration time points, and such data would have provided important insight into phase-specific biodistribution and mechanisms of action. In our previous reports, intravenously administered AMSCs given on Day 1 were predominantly detected in the lung and liver, with minimal evidence of sustained engraftment within the spinal cord, and were largely undetectable three days after transplantation. It is plausible that cells transplanted on Day 3 may undergo even more rapid clearance within the pro-inflammatory and pro-coagulant milieu characteristic of that time window. Second, we primarily used CD68 and Iba1 as markers of macrophage/microglial activation. While these markers reliably indicate myeloid activation, they do not permit definitive discrimination between M1-like and M2-like polarization states, nor do they distinguish resident microglia from infiltrating monocyte-derived macrophages. Therefore, the present study cannot determine whether AMSC therapy induces specific phenotype switching (e.g., pro-inflammatory to reparative polarization) or selectively alters cellular origin. However, our central conclusion concerns the temporal dominance of major inflammatory compartments rather than fine-grained polarization states. Future studies incorporating phenotype-specific markers such as CD206, Arg-1, iNOS, and TMEM119, as well as lineage-tracing approaches, will be necessary to further refine the mechanistic interpretation of phase-specific immunomodulation. Third, we did not perform multiple injection in this report. Sequential D1 + D7 strategy could theoretically provide broader immune-phase coverage and potentially enhance therapeutic efficacy. Finally, we did not measure where the cells or extracellular vesicles (EVs) go in the body, how long they stay in the target tissue, or how strong their effects are, this limits direct proof of the site of action.

## Conclusion

In conclusion, the efficacy of intravenous AMSC therapy for SCI depends on how well the dosing time matches the immune phase. When treatment is synchronized with the dominant, targetable immune cell population—neutrophils in the acute phase or macrophages/microglia in the subacute phase—functional improvement and attenuation of inflammation are most pronounced. By contrast, during transitional phases (e.g., D3), a single, conventional AMSC dose is unlikely to provide robust benefit. This “phase-matching” framework offers a practical path to optimize clinical dosing windows for MSC-based therapies and lays experimental groundwork for biomarker-guided, sequential interventions in personalized regenerative immunotherapy.

## Supplementary Information

Supplementary Material 1.

## Data Availability

All additional files are included in the manuscript. The data that support the findings of this study are available from the corresponding author, [MK], upon reasonable request. Mass spectrometry proteomic data were deposited in the ProteomeXchange Consortium through the jPOST repository with the dataset identifier JPST004295/PXD072646.
