# Supplementary material for: Phase-dependent efficacy of intravenous amniotic mesenchymal stem cells in a rat spinal cord injury model
Source: Stem Cell Res Ther. 2026 Apr 24;17:208. doi: 10.1186/s13287-026-05018-0 (PMC13255217; doi:10.1186/s13287-026-05018-0)
Supplement: Supplementary file 1 — Supplementary Material 1. [file 13287_2026_5018_MOESM1_ESM.docx]

Rat spinal cord injury model

The clip blades were polished to provide a flat contact surface, and the closing force was set to 30 g. Rats were anesthetized with 2–5% isoflurane in 30% O₂ / 70% N₂O, and core temperature was maintained at 36.5–37.5 °C with a heating pad. Animals were placed prone, the T2 spinous process was identified and used to count down to T6–T7, and a laminectomy was performed at T6–T7. The removed laminae were kept in sterile saline. The aneurysm clip was applied extradurally at T6–T7 for 1 min to fully compress the spinal cord, after which the clip was removed, and the laminae were repositioned. The wound was closed, and animals recovered on warmed towels until fully awake. At 24 h post-injury, model success was assessed using the Basso–Beattie–Bresnahan (BBB) score; animals with BBB ≠ 0 were excluded from subsequent studies. Bladders were manually expressed 2–3 times daily until spontaneous reflexive bladder control returned. Animals received chow easily accessible chow and water, with daily welfare checks.

AMSC preparation and cell transplantation

Frozen human AMSC vials were supplied by Kaneka Corporation (Osaka, Japan). Vials were rapidly thawed in a temperature-controlled water bath. For cell counting and viability assessment, 2 µL acridine orange/propidium iodide (AO/PI) staining solution was added to 18 µL of cell suspension, mixed, and 10 µL was loaded onto a LUNA™ Cell Counting Slide (Logos Biosystems, Korea) for analysis with the LUNA™ FL Dual Fluorescence Cell Counter (Logos Biosystems, Korea). Cells were then seeded into polystyrene CellSTACK® culture chambers (REF 3268; Corning, USA) at 1,000 cells/cm². After verifying attachment and morphology by microscopy, cultures were maintained at 37 °C in a CO₂ incubator. On day 6 after seeding, cell suspensions were prepared for transplantation. Cells were detached, passed through a 42 µm filter, and counted again using the same AO/PI method. The suspension was diluted with sterile normal saline to a target concentration of 1 × 10⁶ cells/mL. A confirmatory count was performed; preparations deviating by more than ±5% from the target concentration were adjusted accordingly. Cell suspensions were kept on ice and used immediately for intravenous administration. For transplantation, general anesthesia was induced as described above. Animals were randomly assigned to experimental groups and received either AMSC suspension or vehicle (normal saline) via the lateral tail vein at an injection rate of 1 mL/min. Animals were euthanized at designated time points. Transcardial perfusion was performed via the left ventricle with 200 mL sterile normal saline followed by formalin; the vertebral column was then dissected and immersed in 10% formalin for 48 h for subsequent histological evaluation.

Neurological and histological evaluation

Animals were evaluated in a walled open-field arena. Tests were conducted at the same time of day under stable conditions (quiet room, ~22–24 °C, constant illumination). Before scoring, each rat was placed in the arena for brief acclimation, then allowed to move freely for a 5-min observation period. Two trained observers, blinded to group allocation, independently scored each animal; the final BBB score was the mean of the two ratings.

Paraffin-embedded sagittal sections (6 µm) were cut, mounted on slides, and dried overnight at 40 °C. After deparaffinization and rehydration, sections were incubated overnight at 50 °C in 0.1% LFB in 95% ethanol containing 0.05% acetic acid, then differentiated in 0.05% lithium carbonate (10 s) and 70% ethanol until gray–white matter contrast was clear (white matter blue, gray matter pale), followed by a distilled-water rinse. Sections were counterstained with 0.1% cresyl violet for 6 min, lightly differentiated in 95% ethanol (with 0.1% acetic acid if needed), dehydrated, cleared in xylene, and mounted with a resin medium. Images were acquired using a KEYENCE BZ-X710 microscope (Keyence, Osaka, Japan) under consistent conditions. Lesion length measurements were performed using ImageJ software (version 1.53k, National Institutes of Health, USA, https://imagej.nih.gov/ij/). To characterize immune‐cell regulation in the spinal cord after SCI and at defined treatment time points, we performed immunohistochemistry with spatial and quantitative analysis. Rats were euthanized at: day 28 post-SCI for overall treatment assessment (n = 9–11 per group); days 1, 3, and 7 post-SCI for neutrophil/macrophage dynamics (n = 3 per group); and, for treatment-window validation, neutrophils on the day 1 after AMSC administration and macrophages on day 7 after AMSC administration (n = 4–5 per group). Longitudinal (sagittal) sections (6 µm) were cut on a Leica RM2125 manual rotary microtome (Leica Microsystems, Wetzlar, Germany). Sections were deparaffinized in xylene, rehydrated, and subjected to heat-induced epitope retrieval in either pH 6.0 citrate or pH 9.0 buffer (170 °C for 3 min in a pressure-heated chamber), then blocked for 30 min to reduce non-specific binding. Primary antibodies were incubated 60 min at room temperature (RT): mouse anti-CD68 (Bio-Rad, Cat. 159320, 1:1000), rabbit anti-Iba1 (Fujifilm Wako, Cat. CAP4688, 1:1500), and rabbit anti-myeloperoxidase (MPO) [EPR20257] (Abcam, ab208670, 1:1000). After washing, the Histofine® Simple Stain Rat MAX-PO (MULTI) (Nichirei Biosciences Inc., Tokyo, Japan; Cat. No. 414191) was applied for 30 min at RT per the manufacturer’s instructions; chromogenic development and counterstaining followed standard protocols. Negative controls (no-primary and isotype) were included in each run. Images were acquired on a KEYENCE BZ-X710 microscope (Keyence, Osaka, Japan) at 400× total magnification (40× objective) under identical exposure/white-balance settings, sampling regions ±1 mm and ±5 mm from the lesion epicenter. For each section, five random, non-overlapping fields were recorded. Positive cells were quantified with BZ-X Analyzer (Hybrid Cell Count, Keyence, Osaka, Japan) under fixed thresholds.

Complete blood count (CBC) and systemic immune-inflammation index (SII)

25 rats underwent SCI on day 0 (D0) and were randomized into five groups (n = 5/group; Control group, vehicle group, D1 group, D3 group and D7 group); Operators and analysts were blinded to group allocation. A BBB=0 confirmation was performed at 24 h post-injury (D1, a.m.) before group assignment and the first AMSC/vehicle administration. Longitudinal blood sampling was scheduled at D1, D2, D3, D4, D7, D8, and D14 (a.m.); AMSC (or vehicle) injections were performed D1 (p.m.), D3 (p.m.), and D7 (p.m.) as described in the transplantation section.

For blood collection, ~100 µL of whole blood was collected by a tail-vein nick (right lateral tail). Blood for hematology was drawn into EDTA-coated microcapillaries (TGK TryWinZ hematocrit capillaries with EDTA; Tokyo Glass Kikai, Japan). Samples were analyzed within 2 h of collection (or stored at 4 °C ≤6 h if immediate analysis was not feasible). CBCs and multi-part leukocyte differentials were measured on an automated hematology analyzer (Sysmex XN-2000, WDF channel; Sysmex Corporation, Kobe, Japan) configured for rat blood. Per manufacturer documentation, the WDF channel principle was used; reagents included CellPack DCL (isotonic diluent), Lysercell WDF, and Fluorocell WDF. For each run, 20 µL of whole blood was mixed with 120 µL CellPack DCL and measured in 7× dilution mode. Reagent lots were: CellPack DCL A3385 and A4029, Lysercell WDF A3012, and Fluorocell WDF A3044. The systemic immune-inflammation index was computed from absolute counts as SII = (platelet count × neutrophil count) / lymphocyte count.[31] Samples with invalid or zero lymphocyte counts were excluded from SII calculation.

Data Independent Acquisition (DIA)‑based quantitative blood plasma proteomics

To profile post-injury plasma proteome dynamics in our SCI model and to explore potential bases for time-dependent AMSC effects, DIA-MS was performed at days 1, 3, and 7 after injury. Rats with SCI (n = 6, longitudinal sampling) were bled on days 1, 3, and 7 using the method described above; plasma was prepared promptly at 4 °C by a double-spin protocol (1,000 × g for 10 min, twice; then 2,500 × g for 10 min, once) on a refrigerated microcentrifuge Eppendorf 5417R (Eppendorf AG, Hamburg, Germany), aliquoted to minimize freeze–thaw cycles, and stored at −80 °C.

Samples were digested using the SP3 method as previously described.[32, 33] A TripleTOF 7600 mass spectrometer (Sciex, Framingham, MA, USA) coupled with the Dionex Ultimate 3000 RSLCnano System (Dionex, Sunnyvale, CA, USA) was used for DIA-MS. The peptides and proteins were identified and quantified using DIA-NN 1.8.1 with UniProt rat reference proteome data and filtering at a false discovery rate of < 1%. Normalized protein-abundance matrices were analyzed in RNAseqChef. (https://imeg-ku.shinyapps.io/RNAseqChef_imeg/) [34] Differential expression analysis between the 1st, 3rd, and 7th days group after injury was conducted using the limma package for normalized count data, as implemented in RNAseqChef. Given the exploratory nature of this study, proteins with false discovery rate (FDR) less than 0.1 were considered as significantly differentially expressed; among these, fold change ≥ 1.20 defined upregulation and ≤ 0.83 defined downregulation. Differentially expressed proteins were submitted to STRING v12.0 (https://string-db.org/) for protein–protein interaction network construction and enrichment.[35]
